# Lysosomal dysfunction and autophagy blockade contribute to IMB-6G-induced apoptosis in pancreatic cancer cells

**DOI:** 10.1038/srep41862

**Published:** 2017-01-31

**Authors:** Lu Liu, Na Zhang, Yueying Dou, Genxiang Mao, Chongwen Bi, Weiqiang Pang, Xiaojia Liu, Danqing Song, Hongbin Deng

**Affiliations:** 1 Institute of Medicinal Biotechnology, Chinese Academy of Medical Sciences & Peking Union Medical College, Beijing 100050, China; 2 Zhejiang Provincial Key Laboratory of Geriatrics & Geriatrics Institute of Zhejiang Province, Zhejiang Hospital, Hangzhou, Zhejiang 310013, China

## Abstract

Targeting the autophagic pathway is currently regarded as an attractive strategy for cancer drug discovery. Our previous work showed that IMB-6G is a novel *N*-substituted sophoridinic acid derivative with potent cytotoxicity against tumor cells, yet the effect of IMB-6G on autophagy and pancreatic cancer cell death remains unknown. Here, we show that IMB-6G inhibits the growth of MiaPaCa-2 and HupT-3 pancreatic cancer cells and induces caspase-mediated apoptosis, which is correlated with an accumulation of autophagic vacuoles. IMB-6G promotes autophagosome accumulation from the early stage of treatment but blocks autophagic flux in the degradation stage, mainly through attenuation of lysosomal cathepsin activity in pancreatic cancer cells. Moreover, IMB-6G triggers lysosomal membrane permeabilization (LMP), followed by cathepsin B/CTSB and cathepsin D/CTSD release from lysosomes into the cytoplasm. Inhibition of autophagosome formation with siRNA against autophagy protein 5 (Atg5) attenuates IMB-6G-induced LMP and apoptosis. Furthermore, cathepsin inhibitors relieve IMB-6G-induced apoptosis as well. Altogether, our findings demonstrate that IMB-6G is a novel autophagy inhibitor, which induces autophagy-dependent apoptosis through autophagosomal-cathepsin axis in pancreatic cancer cells and indicate the potential value of IMB-6G as a novel antitumor drug candidate.

Pancreatic cancer is the fourth leading cause of cancer-related deaths worldwide and one of the most aggressive and devastating human malignancies^1^. The overall 5-year survival rate is 3~5% because of advanced stage at diagnosis and poor response to current treatment^2^. Little improvement has been achieved in the treatment of pancreatic cancer for the past 20 years. Therefore, the identification of effective targets and novel therapeutic strategies to improve the outcome in this deadly disease are urgently needed^3^.

Pancreatic tumor has higher basal level of autophagy as compared to other epithelial tumors^4^. Autophagy is a highly conserved process by which, cells get rid of unwanted cellular components including damaged organelles, lipid vesicles, or protein aggregates through lysosomal degradation^5^. It is a dynamic process in which, useless cellular components gets sequestered in double-membranous vesicles called autophagosomes. Autophagosomes fuses with lysosomes to generate autolysosomes, in which the autophagic cargo is degraded by acidic hydrolases and released into the cytosol^6^. Several published studies demonstrated that autophagy can contribute to pancreatic tumor growth and development^7^,^8^. Inhibition of autophagy in pancreatic tumor cells augments production of reactive oxygen species, increases DNA damage, and limits effective metabolism^8^. Furthermore, chloroquine, an anti-malaria drug with autophagy inhibition activity, suppressed pancreatic tumor growth in xenograft mice^9^.

Elevated autophagy in pancreatic cancer is also shown to participate in tumor dormancy and chemoresistance^10^. It has been reported that anti-cancer agents like gemcitabine induce autophagy in pancreatic cancer cells and prevent them from entering the apoptotic pathway^10^. Pancreatic cancer shows initial sensitivity to gemcitabine therapy but rapidly develops resistance^11^, suggesting that inhibition of autophagy might overcome gemcitabine resistance in pancreatic cancer cells. Therefore, targeting the autophagy process in pancreatic cancers may have considerable efficacy for disease treatment.

Natural products provide a bountiful source of new chemotherapeutics. Sophoridine, one of the major bioactive components extracted from the traditional medicine herb *Sophora alopecuroides* L., has been approved by China FDA (CFDA) in 2005 to cure the cancer patients with malignant trophoblastic tumors^12^,^13^. Recently we reported that IMB-6G (Fig. 1a), a new *N*-substituted sophoridinic acid derivative exhibits a potent antiproliferation effect in a panel of human tumor cell lines via inducing G_0_/G_1_ cell cycle arrest and endoplasmic reticulum (ER) stress^14^,^15^. Furthermore, IMB-6G also owns several druggable advantages such as flexibility structure, reasonable bioavailability, favorable pharmacokinetic property and good safety profiles^14^,^16^. However, the effect of IMB-6G on autophagy and pancreatic cancer cell death remains to be determined.

In this report, we aimed to investigate the potential of IMB-6G to modulate autophagy and the underlying mechanism of IMB-6G against human pancreatic cancer. Our results indicated that IMB-6G promoted autophagosome accumulation from the early stage of treatment but blocked autophagic flux through attenuation of lysosomal cathepsins activity in pancreatic cancer cells. This novel autophagy inhibitor eventually induced cathepsin releasing from lysosomes into the cytoplasm and a caspase-mediated apoptotic cell death. These findings have important clinical implications and provide a mechanistic rationale for the use of IMB-6G for the treatment of pancreatic cancer.

## Results

### IMB-6G induces cytotoxicity and caspase-mediated apoptosis in pancreatic cancer cells

**Figure 1:**
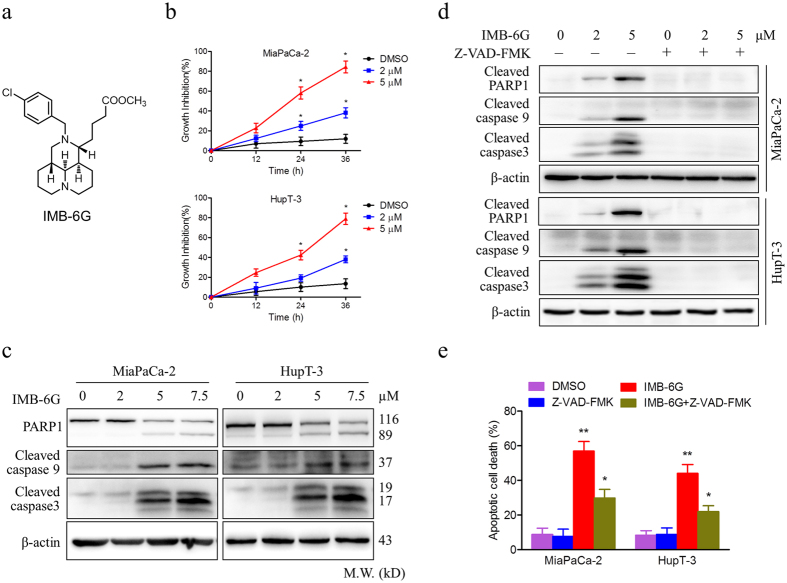
IMB-6G induces cytotoxicity and apoptosis in pancreatic cancer cells. (**a**) Structure of the IMB-6G molecule. (**b**) MiaPaCa-2 and HupT-3 cells were treated with various concentrations of IMB-6G for the indicated time. Cell viability was measured by MTT assay. (**c**) MiaPaCa-2 and HupT-3 cells were treated with indicated concentrations of IMB-6G for 24 h. The protein expression levels of cleaved caspase 9, cleaved caspase 3, and poly (ADP-ribose) polymerases (PARP) were detected by immunoblotting. (**d**) MiaPaCa-2 and HupT-3 cells were treated with IMB-6G in the presence or absence of Z-VAD-FMK (20 μM). Cleaved PARP1, caspase 9 and caspase 3 were detected by immunoblotting. (**e**) MiaPaCa-2 and HupT-3 cells were stained with Annexin V-FITC and PI after incubated with indicated concentrations of IMB-6G or DMSO in the presence or absence of Z-VAD-FMK for 24 h, the numbers of apoptotic cells were analyzed by flow cytometry. Annexin V-positive cells were accepted as apoptotic cells. The results are presented as mean ± SD and represent three individual experiments. *p < 0.05, **p < 0.01 compared with the untreated control group.

To investigate the antitumor activity of IMB-6G on pancreatic cancer, we used methylthiazol tetrazolium (MTT) assay to analyze the effects of IMB-6G on the viability of pancreatic cancer cells. Treatment of MiaPaCa-2 and HupT-3 cells with IMB-6G resulted in significantly increased growth inhibition of cells in a concentration and time-dependent manner (Fig. 1b). The IC_50_ of IMB-6G was 4.5 μM in MiaPaCa-2 cells, 6.5 μM in HupT-3 cells, after 24 h of treatment respectively. To examine whether cell apoptosis was involved in IMB-6G-induced pancreatic cancer cell death, the apoptotic cell death was investigated by immunoblotting. In both the MiaPaCa-2 and HupT-3 cells, IMB-6G treatment led to significant activation of poly (ADP-ribose) polymerase 1(PARP1), cleaved caspase 9 and caspase 3 in a concentration-dependent manner (Fig. 1c). In addition, Annexin V/propidium iodide (PI) double staining results also indicated that IMB-6G induced phosphatidylserine plasma membrane externalization in MiaPaCa-2 and HupT-3 cells (Supplementary Fig. S1). To elucidate whether caspase activation is required for IMB-6G-induced cell death, we exposed cells to Z-VAD-FMK, a pancaspase inhibitor. Immunoblotting results showed that addition of Z-VAD-FMK completely prevented PARP1, caspase 9 and caspase 3 cleavage induced by IMB-6G (Fig. 1d). Moreover, Flow cytometry results indicated that IMB-6G induced apoptotic cell death was rescued by Z-VAD-FMK in MiaPaCa-2 and HupT-3 cells (Fig. 1e). These results thus demonstrate that IMB-6G induces cytotoxicity and caspase-mediated apoptosis in pancreatic cancer cells.

### IMB-6G triggers autophagosome formation in pancreatic cancer cells

**Figure 2:**
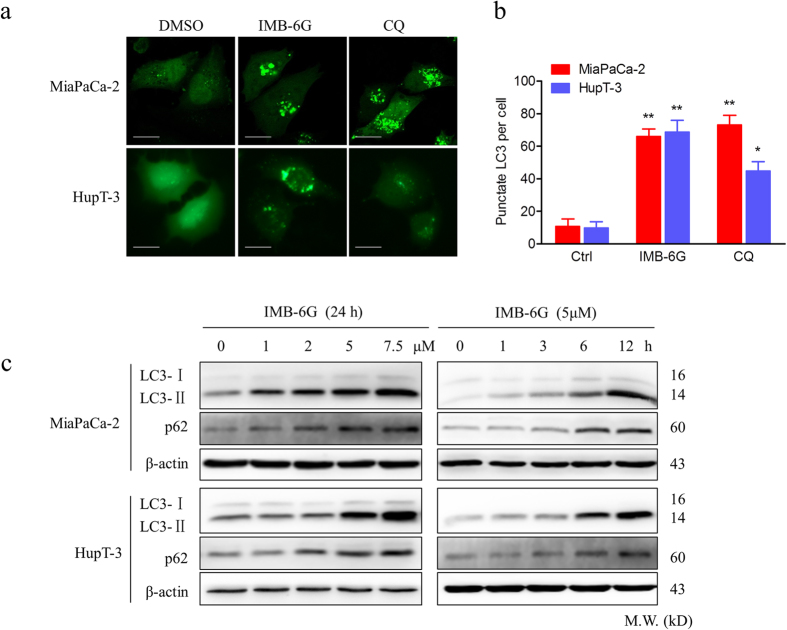
IMB-6G induces autophagosome accumulation in pancreatic cancer cells. (**a**) MiaPaCa-2 and HupT-3 cells were transfected with the EGFP-LC3 plasmid. After 24 h, the cells were incubated with IMB-6G (5 μM), CQ (50 μM) or DMSO (Ctrl) for 6 h and visualized with confocal microscopy (upper panel; scale bars, 20 μm). (**b**) The number of punctate EGFP-LC3 in each cell was counted, and at least 100 cells were included for each group (lower panel). Data were the mean value of three independent experiments with each count of no less than 100 cells.*p < 0.05, **p < 0.01 compared with the untreated control group. (**c**) MiaPaCa-2 and HupT-3 cells were treated with IMB-6G at the indicated concentrations for 24 h, or treated with IMB-6G (5 μM) for indicated time points, the lipidation of LC3 and the levels of p62/SQSTM1 were detected by immunoblotting using corresponding antibodies.

Accumulating evidences indicate that autophagy plays a crucial role in the regulation of apoptosis^17^. To investigate the mechanism by which IMB-6G treatment induced apoptosis, we determined whether IMB-6G could modulate autophagy in pancreatic cancer cells. We transfected MiaPaCa-2 and HupT-3 cells with the enhanced green fluorescent protein-microtubuleassociated protein 1 light chain 3 (EGFP-LC3) reporter, as a specific indicator of autophagic vacuoles. The redistribution of EGFP-LC3 from cytosol to autophagosome indicates the formation of autophagosomes. In MiaPaCa-2 and HupT-3 cells expressing EGFP-LC3, IMB-6G treatment for 6 h increased numbers of fluorescent puncta per cell, similar to that seen following Chloroquine (CQ) treatment (Fig. 2a,b), CQ is a widely used autophagy inhibitor. By contrast, control cells showed a relative diffuse green fluorescence in the cytosol (Fig. 2a). To further confirm the formation of autophagic vesicles, we next explored the LC3-II conversion, which participates in elongation of the autophagosome membrane^18^. As expected, LC3-II levels in MiaPaCa-2 and HupT-3 cells were increased in a concentration and time-dependent manner (Fig. 2c, upper panel). Notably, IMB-6G increased the expression of LC3-II as early as 6 h of treatment, indicating IMB-6G increased the autophagysome formation from the early stage of treatment. Taken together, these results demonstrated that IMB-6G treatment resulted in autophagosome accumulation in pancreatic cancer cells.

### IMB-6G inhibits autophagic flux in the degradation stage of autophagy

**Figure 3:**
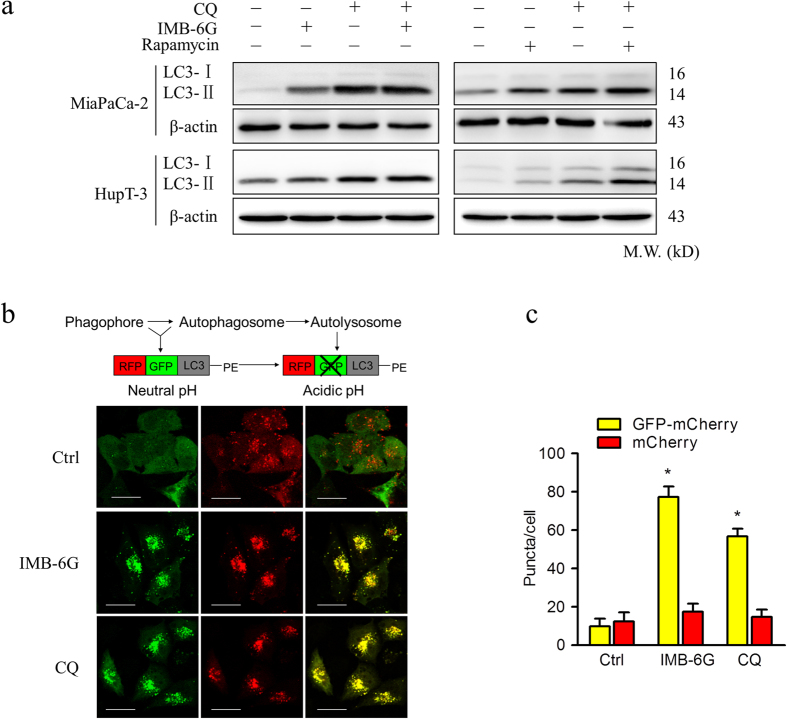
IMB-6G blocks autophagic flux in the degradation stage. (**a**) MiaPaCa-2 and HupT-3 cells were pretreated with CQ (50 μM) or rapamycin (200 nM) for 2 hours, followed by IMB-6G (5 μM) treatment for 12 h. The LC3 turnovers in both cells were detected by immunoblotting. (**b**) MiaPaCa-2 cells were transfected with mCherry-EGFP-LC3 plasmid, followed by treatment with IMB-6G (5 μM) or CQ (50 μM) for 6 h. Representative fluorescent images are visualized with confocal microscopy (scale bars, 20 μm). (**c**) Quantification of GFP/mCherry double-positive and mCherry single-positive puncta per cell in control or cells treated with CQ or IMB-6G. Data were the mean value of three independent experiments with each count of no less than 100 cells. Values are expressed as the mean ± SD, *p < 0.05, **p < 0.01 vs. untreated control.

The amount of autophagosomes in the cytosol can be increased by two different mechanisms: increased autophagosome synthesis by upstream processes and blockade of lysosomal degradation at a later stage. To discriminate between these two possibilities, we measured the effect of IMB-6G on autophagic flux. This was accomplished by measuring the total amount of p62/sequestosome-1(SQSTM1), which has been implicated in autophagic cargo recognition and is lost in the final stages of autophagy during autolysosome degradation^19^. An increase in the amount of p62/SQSTM1 is related to the inhibition of autophagy flux. Immunoblotting results revealed that p62/SQSTM1 levels increased in a concentration- and time-dependent manner in IMB-6G-treated MiaPaCa-2 and HupT-3 cells (Fig. 2c, middle panel), indicating IMB-6G induced accumulation of autophagosomes reflects an inhibition of their degradation in pancreatic cancer cells. This phenomenon was further confirmed by the LC3-II turn over assay^19^. MiaPaCa-2 and HupT-3 cells were treated with IMB-6G (5 μM) in the presence or absence of CQ (50 μM) for 2 h. To mimic autophagy induction, we treated cells with rapamycin (200 nM) as a control. If the autophagy is induced, co-treatment with CQ will increase the LC3-II level. On the contrary, LC3-II level will not be affected in the presence of CQ. As expected, we observed that CQ treatment had no effects on LC3-II levels in IMB-6G-treated cells (Fig. 3a, left panel). By contrast, in rapamycin-treated cells, co-treatment with CQ increased LC3-II levels (Fig. 3a, right panel).

To further investigate the role of IMB-6G in blocking autophagic flux in pancreatic cancer cells, we transfected MiaPaCa-2 cells with a plasmid encoding membrane-localized red fluorescent protein mCherry-EGFP-LC3. mCherry retains its fluorescence even in the acidic environment of lysosomes, whereas GFP fluorescence is easily quenched in a low-pH environment^20^. The red puncta that overlayed with the green puncta (merged as yellow) were thus indicators of autophagosomes that are not fused with acidic lysosomes, whereas the solely red puncta were indicative of autolysosomes^20^. Therefore, both yellow and red punctate fluorescence will increase in the case of autophagy activation, whereas blockade in the degradation stage results in only yellow punctate fluorescence^20^. After treatment of the cells with IMB-6G (5 μM) for 6 h, only yellow punctuate fluorescence was markedly increased, similar to that seen following CQ treatment, indicating a blockade of autophagy in IMB-6G-treated cells (Fig. 3b,c). Together, these results demonstrated that IMB-6G-induced autophagosome accumulation was due to impaired autophagic flux, indicating that IMB-6G was a potent autophagy inhibitor.

### IMB-6G blocks autophagic flux through attenuation of lysosomal cathepsin activity

**Figure 4:**
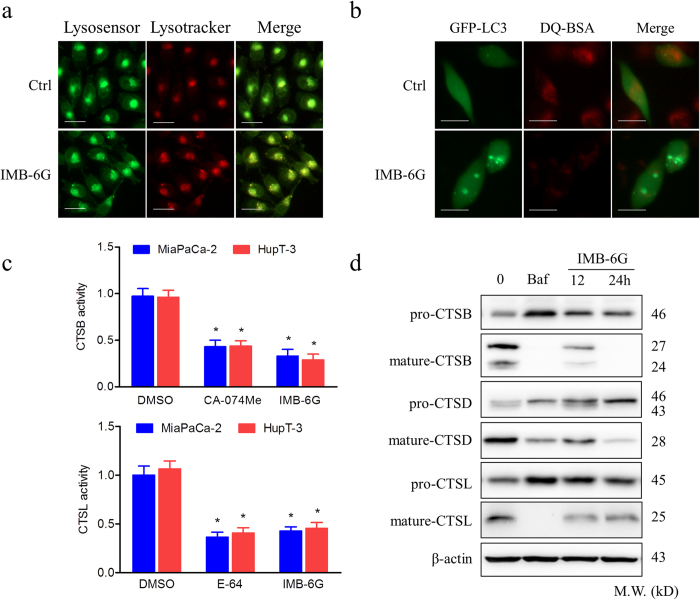
IMB-6G blocks autophagic flux through attenuation of cathepsin activity in pancreatic cancer cells. (**a**) MiaPaCa-2 cells were incubated with IMB-6G (5 μM) for 12 h, followed by staining with LysoTracker Red (100 nM) for 30 min, cells were incubated with a LysoSensor Green dye (2 μM) for 10 min. Merged (yellow) images are indicative of an acidic pH (scale bars, 20 μm). (**b**) MiaPaCa-2 cells were transfected with the EGFP-LC3 plasmid. After 24 h, the cells were treated with IMB-6G (5 μM) for 12 h before being incubated with DQ-Red BSA (10 μg/ml) for 30 min. The cells were fixed and analyzed for fluorescence microscopy (scale bars, 20 μm). (**c**) Enzymatic activity of CTSB and CTSL in IMB-6G treated MiaPaCa-2 and HupT-3 cells. Cells were treated with DMSO, IMB-6G (5 μM), CA-074Me (2 μM, CTSB inhibitor) or E-64 (10 μM, CTSB and CTSL inhibitor) for 24 h. Enzymatic activity was analyzed using fluorogenic kits. Data are presented as the mean ± SD from 3 independent experiments. (**d**) MiaPaCa-2 cells were treated with 5 μM IMB-6G for 12 or 24 h, the precursor and the mature form of CTSB, CTSD and CTSL were determined by immunoblotting. Bafilomycin (Baf, 200 nM) was used as a positive control.

IMB-6G inhibited the degradation stage of autophagy indicated it may be involved in the function of the lysosome. To this end, we examined the effects of IMB-6G on lysosomal function. First, because acidic pH is required for lysosomal activity^21^, we used LysoSensor Green dye staining to evaluate whether IMB-6G affected lysosomal pH. MiaPaCa-2 cells were stained with lysotracker red fluorescent dye to measure acidic organelles and lysosenser green dye to measure pH, the merged yellow fluorescence images thus indicate an acidic environment. As shown in Fig. 4a, IMB-6G produced an accumulation of acidic vesicles as compared with DMSO-treated control cells, indicating that impaired autophagic flux induced by IMB-6G was not due to inhibition of lysosomal acidification. Next, to monitor lysosomal activity during IMB-6G treatment, MiaPaCa-2 cells were assayed for their ability to process DQ-BSA (a derivative of BSA), the red fluorescence of which is quenched unless it is cleaved by proteolytic enzymes^22^. As shown in Fig. 4b, very little dequenching of DQ-BSA occurred in IMB-6G-treated cells, indicating that intracellular proteolytic activity was inhibited in the presence of IMB-6G. Thus, we next assessed whether cathepsin activity was attenuated by IMB-6G by measuring the enzymatic activity of Cathepsin B (CTSB) and L (CSTL) in MiaPaCa-2 and HupT3 cells. Both CTSB and CTSL activities were reduced following IMB-6G treatment, similar to that seen following CA-074e (a specific CTSB inhibitor) and E-64 (CTSB and CTSL inhibitor) treatment, indicating that IMB-6G significantly inhibits the activity of cathepsins (Fig. 4c). Without a specific substrate, activity of CTSD could not be determined. Furthermore, we investigated the effects of IMB-6G on cathepsin processing by immunoblotting using antibodies recognizing the preform and the mature form of CTSB, CTSD and CTSL. Consistently, IMB-6G significantly impaired the maturation of CTSB, CTSD and CTSL (Fig. 4d). Taken together, our data demonstrated that IMB-6G impairs autophagic flux by inhibiting lysosomal cathepsin activity.

### IMB-6G induced autophagy-dependent lysosomal membrane permeabilization (LMP)

**Figure 5:**
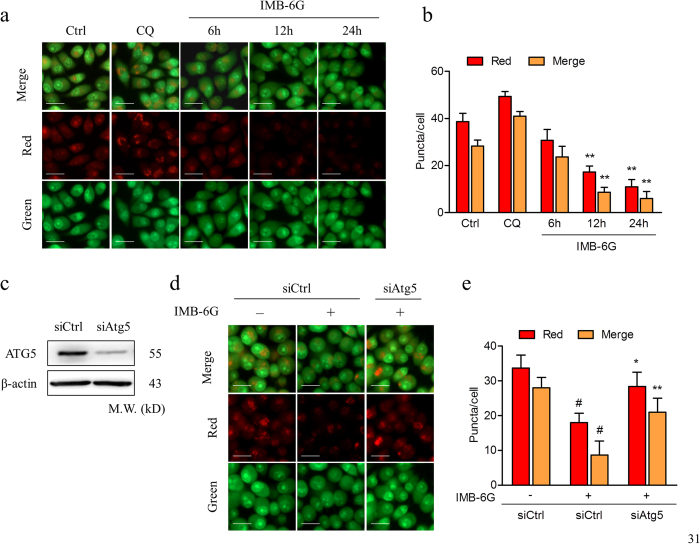
IMB-6G induced autophagy-dependent LMP. (**a**) MiaPaCa-2 cells were treated with 2 μM IMB-6G for 6 h, 12 h, or 24 h. Lysosomal membrane stability was measured by AO staining under a fluorescence microscopy. CQ (50 μM) was used as a positive control (scale bars, 20 μm). (**b**) Quantification of red and yellow fluorescence intensity of AO in MiaPaCa-2 cells. Data were the mean value of three independent experiments with each count of no less than 100 cells. Values are expressed as the mean ± SD, **p < 0.01 vs. untreated control. (**c–e**) MiaPaCa-2 cells were transfected with either 50 nM siRNA against human Atg5 (siAtg5) or control siRNA (siCtrl), and then treated with 5 μM IMB-6G for 12 h. (**c**) siRNA transfection efficiency was assessed by immunoblotting after 48 h of transfection. β-actin was used as an internal control. (**d**) Lysosomal membrane stability was measured by AO staining under a fluorescence microscopy (scale bars, 20 μm). (**e**) The quantification of red and yellow fluorescence intensity of AO was shown. Values are expressed as the mean ± SD, ^#^*p* < 0.01 compared with IMB-6G-untreated siCtrl group, *p < 0.05, **p < 0.01 compared with IMB-6G-treated siCtrl group.

Because IMB-6G disrupts lysosomal degradation to inhibit the autophagy process, we next focused on examining the effect of IMB-6G on lysosomal integrity. We performed acridine orange (AO) staining to examine lysosomal membrane stability. As shown in Fig. 5a and b, after treatment with 5 μM IMB-6G for 12 h and 24 h, the numbers of red puncta (intact lysosome) was significantly decreased as compared with the control. This suggests IMB-6G induced significant LMP in MiaPaCa-2 cells. After treatment with 5 μM IMB-6G for 6 h, the intensity of red fluorescence did not change significantly as compared with the control (Fig. 5a,b). Taken together with the evidence of early increased expression of LC3-II (Fig. 2c), these data suggested that IMB-6G-activated autophagosome formation preceded LMP. To further confirm the role of autophagy in IMB-6G-induced LMP, MiaPaCa-2 cells were pretreated with siRNA against autophagy protein 5 (Atg5), a protein necessary for the formation of preautophagosomal structure^19^. Knockdown of Atg5 attenuated IMB-6G-induced LMP in MiaPaCa-2 cells (Fig. 5c–e). These data indicated that IMB-6G-induced LMP was autophagy dependent.

### Inhibition of autophagosome formation alleviated IMB-6G-induced lysosomal release of cathepsin

**Figure 6:**
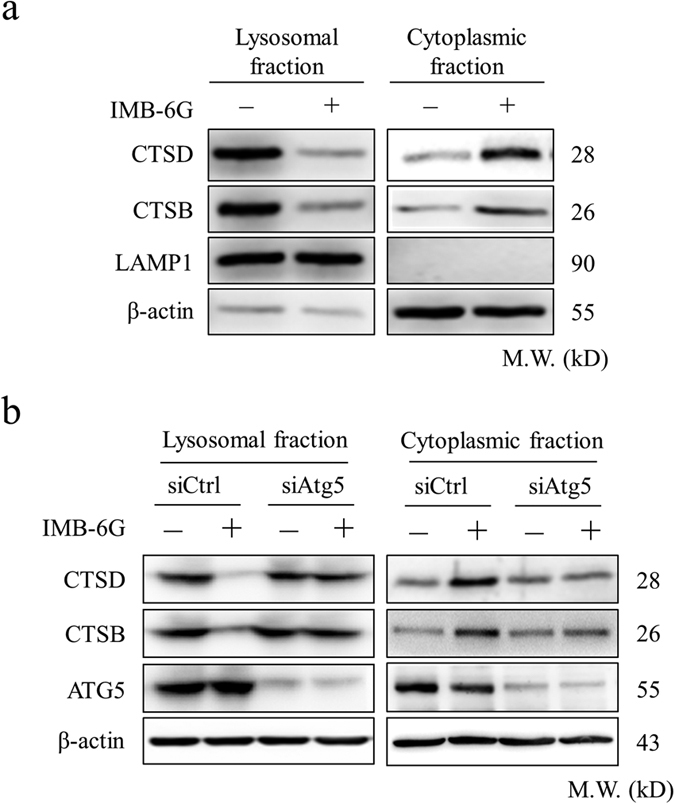
Inhibition of autophagosome formation alleviated IMB-6G-induced lysosomal release of cathepsin. (**a**) MiaPaCa-2 cells were treated with 5 μM IMB-6G for 12 h, cell fragments were performed to separate lysosomal and cytosolic fraction from DMSO- or IMB-6G-treated cells. CTSB and CTSD levels were detected by immunoblotting of the different fractions. Lysosomal-associated membrane protein 1(LAMP1) was as a lysosomal marker, β-actin was a cytosolic marker. (**b**) MiaPaCa-2 cells were transfected with control siRNA or siAtg5, followed by IMB-6G treatment for 12 h, lysosomal and cytosolic fraction were separated and CTSB, CTSD levels were determined by immunoblotting.

The lysosomal aspartic protease cathepsin is a key mediator of apoptosis and its release into the cytosol has been reported to promote cell death^23^,^24^. To further determine whether the reduction in the acidic compartment following IMB-6G treatment was due to lysosomal permeabilization, the localization of CTSB and CTSD was analyzed by immunoblotting. Treatment with IMB-6G for 12 h caused a decrease in CTSB and CTSD in the lysosomal fraction and a concomitant increase in the cytoplasmic fraction (Fig. 6a). After treatment with 5 μM IMB-6G for 6 h, the CTSB and CTSD levels in lysosomal and cytoplasmic fraction did not change significantly as compared with the control (data not shown). These results were consistent with the data of IMB-6G-induced LMP (Fig. 5a,b). Additionally, knocking down Atg5 attenuated IMB-6G-induced lysosomal release of CTSB and CTSD in MiaPaCa-2 cells (Fig. 6b). These data thus suggests that inhibition of autophagosome formation alleviated IMB-6G-induced lysosomal release of cathepsin.

### Blunting autophagosome formation and cathepsin activity protect cells against IMB-6G-induced cell death

**Figure 7:**
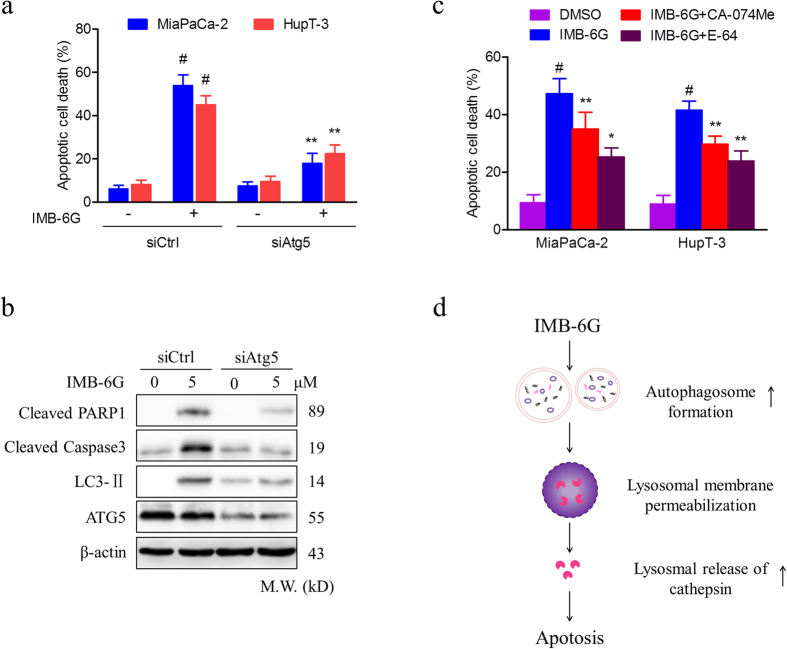
Blunting autophagosome formation and cathepsin activity protects cells from IMB-6G-induced cell death. MiaPaCa-2 and HupT-3 cells were transfected with control siRNA or siAtg5, followed by IMB-6G (5 μM) treatment for 24 h. (**a**) Apoptotic cell death was measured by flow cytometry. Values are expressed as the mean ± SD, ^#^*p* < 0.01 compared with IMB-6G-untreated siCtrl group, **p < 0.01 compared with IMB-6G-treated siCtrl group. (**b**) Cleaved PARP1/caspase3, LC3-II, and ATG5 were detected by immunoblotting. (**c**) MiaPaCa-2 and HupT-3 cells were treated with CA-074Me (CTSB specific inhibitor) or E-64 (CTSB and CTSL inhibitor), followed by IMB-6G (5 μM) treatment for 24 h, the apoptotic cell death was measured by flow cytometry. Data are mean ± SD of 3 independent experiments, ^#^*p* < 0.01 compared with DMSO-treated group, *p < 0.05, **p < 0.01 compared with IMB-6G-treated group. (**d**) The proposed pathway of IMB-6G-induced autophagy-dependent apoptosis in pancreatic cancer cells. IMB-6G-activated autophagosome formation is an upstream event that may trigger LMP. IMB-6G-induced LMP causes lysosomal release of cathepsin and eventually triggers apoptosis.

To further explore the link between the inhibition of autophagy and cell viability by IMB-6G, we used siRNA against Atg5 to determine IMB-6G-induced apoptosis in pancreatic cancer cells. If IMB-6G-induced cell death is due to the induction of autophagosomes, silencing of Atg5 could be protective. Knocking down of Atg5 reduced IMB-6G-induced autophagosomal formation in MiaPaCa-2 cells (Supplementary Fig. S2). Importantly, silencing of Atg5 obviously inhibited IMB-6G-induced apoptotic cell death (Fig. 7a) and significantly rescued IMB-6G-induced cleavage of PARP1 and caspase3 (Fig. 7b). These results suggest that constitutive autophagy contributed to IMB-6G-induced cell death.

As IMB-6G induces lysosomal permeabilization and release of CTSB/CTSD (Figs 5 and 6), the role of cathepsins in IMB-6G-induced cell death was explored using chemical inhibitors of cathepsins. As shown in Fig. 7c, both CA-074Me (CTSB inhibitor) and E-64 (CTSB and CTSL inhibitor), partially prevented IMB-6G-induced cell death in MiaPaCa-2 cells, indicating that cathepsins might partially involve in IMB-6G-induced cell death. Taken together, these data indicated that autophagosomal-cathepsin axis might play an important role in IMB-6G-induced apoptosis.

## Discussion

Recently, autophagy has emerged as an important target in cancer^25^,^26^. Our previous studies have shown that IMB-6G is a new *N*-substituted sophoridinic acid derivative with potent antitumor activity^14^,^16^,^27^. However, little is known about the ability of IMB-6G to affect autophagy in tumor cells. Our current study established that IMB-6G as a novel autophagy inhibitor. Treatment with IMB-6G leads to substantial accumulation of both LC3-II and p62/SQSTM1 in pancreatic cancer cells. In addition, IMB-6G induces LMP and impairs the maturation of CTSB, CTSD and CTSL. Furthermore, IMB-6G selectively induces growth inhibition and apoptosis in pancreatic cancer cells in a dose-dependent manner (Supplementary Figure S3a,b). Since pancreatic cancer cells are constantly under high basal levels of autophagy, they may be particularly sensitive to autophagy inhibition-induced cell death by IMB-6G compared with normal pancreatic ductal epithelial cells. As such, we identify IMB-6G as a novel autophagy inhibitor with chemical structure distinct from other known inhibitors.

It is well established that autophagy can facilitate cell death or cell survival depending on the nature of stimuli and cellular context^5^. In order to investigate the effect of IMB-6G on regulation of autophagy, we tried to decrease the concentrations of IMB-6G to weaken its effects on lysosomes. But we failed to observe a significant activation of autophagy. However, we found that IMB-6G increase of LC3-II was constitutively accompanied with an increase of p62/SQSTM1 as early as 6 h treatment (Fig. 2c), which indicated the blockade of autophagy by IMB-6G. LC3 turnover assays also confirmed the autophagic flux was indeed blocked by IMB-6G (Fig. 3a). In addition, IMB-6G caused LMP in pancreatic cancer cells after treatment for 12 h, but not 6 h (Fig. 5a). Induction of LMP has been abundantly linked to cell death^28^. This suggests that LMP may have occurred after autophagic flux inhibition in IMB-6G-treated pancreatic cancer cells. Furthermore, knocking down Atg5 alleviated IMB-6G-induced LMP and the abrogation of cathepsin activity by chemical inhibitors attenuated cell death in the presence of IMB-6G (Fig. 7), implying that increased LMP is involved in the death-promoting effects of IMB-6G. Our results support the conclusion that the inhibition of autophagy has a central role in IMB-6G-mediated pancreatic cancer cell death. This finding is in agreement with recent studies which show that disruption of the late stage of autophagy leads to excessive accumulation of autophagic vacuoles and has the potential to turn autophagy into a destructive process^29^,^30^.

Our previous study demonstrates IMB-6G induces ER stress-mediated apoptosis in hepatocellular carcinoma cells^15^. But how IMB-6G induces ER stress remains unclear. ER-associated degradation (ERAD) is a physiological pathway that can regulate ER stress responses as well as in the degradation of the unfolded proteins. The ERAD I is a proteasome/ubiquitination pathway, while the ERAD II pathway is a lysosomal activity pathway^31^,^32^. In order to understand how IMB-6G induces ER stress, we investigated the effect of IMB-6G on proteasomal and lysosomal activity. The results showed that the proteasomal activity was not affected by IMB-6G (data not shown), but IMB-6G significantly decreased the lysosomal activity in pancreatic cancer cells (Fig. 4). Since lysosome-associated protein degradation also functions as a cytoplasmic quality control mechanism to eliminate protein aggregation and damaged organelles, our observation of IMB-6G inhibition of lysosomal activity may explain how it induces ER stress.

The increased levels of CTSB/CTSD in cytosolic extracts confirmed the IMB-6G-induced LMP in pancreatic cancer cells. Elevated levels of cytosolic CTSB/CTSD were detected after treatment with 5 μM IMB-6G for 12 h in the cells (Fig. 6a). Inhibition of autophagosome formation with siRNA against Atg5 attenuated IMB-6G-induced lysosomal release of CTSB/CTSD (Fig. 6b). CTSB/CTSD is an important mediator of apoptosis after LMP^33^. Upon LMP, the released CTSD induces conformational change of Bax and its translocation to the mitochondria, subsequently promoting apoptosis^34^. Previously we demonstrated that IMB-6G induced mitochondrial apoptosis through releasing cytochrome c into the cytosol and Bax translocation into mitochondrial^15^. These data indicated that IMB-6G-induced LMP caused lysosomal release of CTSB/CTSD and eventually triggered mitochondrial apoptosis.

In autophagy, the lysosome is the only way to degrade cargos, so complete inhibition of the lysosome always causes serious impairment of autophagic flux^35^. Our results suggested that IMB-6G severely prevented the activation of lysosomal enzymes and thereby hampered their ability to degrade the cargo of the autolysosome, demonstrating that IMB-6G belongs to the late-stage autophagy inhibitor. Late-stage autophagy inhibitors have proven to be useful adjuvants in many antitumor therapies *in vivo*^36^. On the basis of the upregulation of autophagy in cancer cells treated with chemotherapeutic agents, autophagy inhibition has become a promising strategy for cancer treatment^37^. CQ and its derivatives are widely used as late-stage autophagy inhibitors and have been evaluated in several phase I and II clinical trials in combination with chemotherapy drugs in a variety of tumor types^38^,^39^. Notably, in this study we demonstrated IMB-6G, a natural compound with chemical structure distinct from CQ, as a novel late-stage autophagy inhibitor and lead compound for pancreatic cancer therapy.

In summary, our results showed that IMB-6G is a potent antitumor agent that acts via induction of autophagy-dependent apoptosis in pancreatic cancer cells. IMB-6G- induced autophagosome formation might be an upstream event that trigged lysosomal membrane permeabilization, followed by cathepsins releasing from lysosomes into the cytoplasm, and finally induced caspase-dependent apoptosis (Fig. 7d). Targeting autophagy by IMB-6G may thus provide a novel therapeutic option in the treatment of pancreatic cancer.

## Materials and Methods

### Chemicals and Reagents

IMB-6G was synthesized and dissolved in DMSO as previously described^15^. Rapamycin, LysoTracker Red probe and Lysosenser Green dye were purchased from Invitrogen (Carlsbad, CA, USA). Chloroquine (CQ), Acridine orange (AO) and β-actin antibody were obtained from Sigma-Aldrich (St. Louis, MO, USA). Z-VAD-FMK was purchased from Santa Cruz Biotechnology (Santa Cruz, CA, USA). Antibodies against p62, LC3, ATG5, PARP-1, cleaved caspase9 and cleaved caspase3 were purchased from Cell Signaling Technology (Danvers, MA, USA).

### Cell culture and treatment

MiaPaCa-2 and HupT-3 human pancreatic cancer cell lines were procured from ATCC. The cells were cultured in DMEM (Hyclone, UT, USA) supplemented with 10% fetal bovine serum (Hyclone, UT, USA), 100 U/ml penicillin, and100 μg/ml streptomycin sulfate, and incubated at 37 °C in a humidified atmosphere with 5% CO_2_. All the cells used in this study were within twenty passages after receipt or resuscitation. At about 80% confluency, the cells were treated with indicated concentrations of IMB-6G (dissolved in DMSO) for various periods.

### Plasmids and RNA interference

The EGFP-LC3 plasmid and mCherry-EGFP-LC3 plasmid were kindly provided by Dr. Xuejun Li (Peking University, Beijing, China). To determine the role of autophagy in IMB-6G-induced apoptosis, we used siRNA against Atg5 to block autophagosome formation. Cells were transfected with either 50 nM siRNA against Atg5 or scrambled control siRNA (Gene Pharma, Shanghai, China) using Lipofectamine 2000 (Invitrogen, Carlsbad, CA, USA) according to the manufacturer’s instructions.

### Cell proliferation and apoptosis assay

The effect of IMB-6G on the cell viability of pancreatic cancer cells was evaluated *in vitro* using the MTT assay. Briefly, Cells were seeded (5 × 10^3^/well) into 96-well plate and the investigated compounds were added at indicated concentrations. Next, MTT solution at a concentration of 5 mg/ml was added to each well. After subsequent 4 h, the culture medium was removed and formazan crystals were dissolved with 150 μl DMSO. Finally, the absorbance was measured at 570 nm using a microplate reader (Multiskan FC, Thermo, USA).

To measure apoptosis, an FITC Annexin-V apoptosis detection kit (KeyGen, Nanjing, China) was used according to the manufacturer’s instructions. The cells were harvested and washed twice with PBS, then cells were resuspended in binding buffer and incubated with annexin V-FITC and PI for 30 min in the dark. After that, the fluorescence of each sample was quantitatively analyzed by FACSCalibur flow cytometer and CellQuest software (BD Biosciences, Sparks Glencoe, MD, USA).

### Autophagy assays

To study the effect of IMB-6G on autophagosome formation, MiaPaCa-2 and HupT-3 cells were transiently transfected with the EGFP-LC3 plasmid using Vigofect (Vigorous Biotechnology, China) and incubated for 24 h, followed by IMB-6G treatment and then taking the images by confocal microscopy (Zeiss, LSM710 Germany). The number of EGFP-LC3 puncta per cell was quantificated using Image J, one group included at least 100 cells. To determine the formation of autophagosomes and autolysosomes, mCherry-EGFP-LC3 plasmid was transfected into cells and incubated for 24 h. Images were acquired using confocal microscopy. The number of autophagosomes (number of GFP puncta) and autolysosomes (number of mCherry puncta minus number of GFP puncta) were quantificated per cell, and at least 100 cells were included.

### DQ-Red BSA staining

Lysosomal-dependent proteolysis was visualized with DQ-Red BSA (Molecular Probes/Invitrogen, D-12051) at a concentration of 10 μg/ml for 0.5~1 h (37 °C, 5% CO_2_). The cells were then washed 3 times with PBS before being treated with 5 μM IMB-6G for 12 h. Then cells were observed using confocal microscopy (Zeiss, LSM710, Germany).

### LysoSensor/LysoTracker Red staining

Cells were incubated with a medium containing the specified drugs for the indicated times, and then stained with 2 μM LysoSensor DND-160 and 100 nM LysoTracker Red DND-99 (Invitrogen) for 10~30 min. After washed with probe-free medium, the samples were viewed using fluorescence microscopy (Zeiss, Axio Vert. A1).

### Measurement of lysosomal membrane stability

Lysosomal stability was assessed by the AO-relocation method. AO exhibits red fluorescence at high concentrations (in intact lysosomes), but green fluorescence at low concentrations (when lysosomal contents diffuse into the cytosol)^40^. MiaPaCa-2 cells were seeded out on coverslips in 24-well plates. The cells were treated with 5 μM IMB-6G for 6 h, 12 h, or 24 h. The cells were then stained with 5 μg/ml AO (Amresco, Solon, OH, USA) at 37 °C for 30 min, rinsed twice with ice-cold PBS. Samples were observed under a fluorescence microscopy. Lysosomal stability was assessed by red AO-fluorescence, using Image-Pro plus 6.0 software.

### Cathepsin activity assay

Cathepsin activity was determined using the commercial assay provided by Biovision according to the manufacturer’s protocol. Cells were seeded in six-well plate 24 hours before treatment with various concentrations of IMB-6G or vehicle control. Twenty-four hours posttreatment, cathepsin activity was measured using 10 mmol/L CTSB (Z-Arg-Arg-MCA) or L substrate ((Z-Phe-Arg)_2_-R110). A fluorometer (Berthold LB960, Germany) was used to quantify the cleavage of synthetic substrate of CTSB and CTSL. Cathepsin activity was expressed as relative fluorescence units (RFU) per microgram protein.

### Immunoblotting analysis

Immunoblotting was performed as described previously^12^. Briefly, MiaPaCa-2 and HupT-3 cells were washed with PBS and lysed in M2 lysis buffer (20 mM Tris-HCl, pH 7.5, 150 mM NaCl, 10 mM β-glycerophosphate, 5 mM EGTA, 1 mM sodium pyrophoshate, 5 mM NaF, 1 mM Na_3_VO_4_, 0.5% Triton X-100, and 1 mM DTT) supplemented with protease inhibitor cocktail (Sigma P8340). Proteins were separated by SDS-PAGE and electrically transferred to a polyvinylidene difluoride membrane. The membrane was probed with the appropriate primary antibody and with a HRP-conjugated secondary antibody. Blots were visualized by Tanon 5200 system (Tanon, Shanghai, China).

### Cell fractionation

Lysosomal fractions were extracted from cell homogenates by Lysosome Extraction Kit (Sigma-Aldrich; LYSISO1) according to the manufacturer’s protocol. Briefly, cell homogenates were centrifuged for 10 min at 1000 × g at 4 °C. The supernatant fraction was centrifuged for 20 min at 20,000 × g at 4 °C to pellet lysosomes and other organelles, and the resulting supernatant fraction was collected as cytosolic fraction. The pellet fractions were subjected to additional centrifugation. The final pellet (lysosomal) fraction was lysed in the lysis buffer described in the procedure. Samples were subjected to immunoblotting.

### Statistical analysis

Results are presented as mean values ± standard error of independent triplicate experiments. All statistical analyses were performed by using ANOVA and Dunnett’s post hoc test, and *p*-values of less than 0.05 were considered statistically significant.

## Additional Information

**How to cite this article:** Liu, L. *et al*. Lysosomal dysfunction and autophagy blockade contribute to IMB-6G-induced apoptosis in pancreatic cancer cells. *Sci. Rep.* **7**, 41862; doi: 10.1038/srep41862 (2017).

**Publisher's note:** Springer Nature remains neutral with regard to jurisdictional claims in published maps and institutional affiliations.

## Supplementary Material

Supplementary Information
