# Supplementary material for: Lysosomal dysfunction and autophagy blockade contribute to IMB-6G-induced apoptosis in pancreatic cancer cells
Source: Sci Rep. 2017 Jan 31;7:41862. doi: 10.1038/srep41862 (PMC5282566; doi:10.1038/srep41862)
Supplement: Supplementary Information [file srep41862-s1.pdf]

Supplementary Information

Lysosomal dysfunction and autophagy blockade contribute to IMB-6G-induced apoptosis in pancreatic cancer cells

Lu Liu , Na Zhang , Yueying Dou , Genxiang Mao , Chongwen Bi , Weiqiang Pang , Xiaojia Liu , Danqing Song [*], Hongbin Deng [*]

Supplementary Figure Legends

Supplementary Figure S1. IMB-6G induces apoptosis in MiaPaCa-2 and HupT-3 cells. (**a**) MiaPaCa-2 and HupT-3 cells were stained with Annexin V-FITC and PI after incubated with 3μM of IMB-6G for 24 h, the numbers of apoptotic cells were analyzed in flow cytometry. (**b**) Statistical analysis result of flow cytometric analysis of apoptosis. Annexin V-positive cells were accepted as apoptotic cells. The results are presented as mean ± SD and represent three individual experiments. $^{**}p<0.01$ compared with control group.

Supplementary Figure S2. Silence of Atg5 reduced IMB-6G-induced autophagosomal formation. (**a**) MiaPaCa-2-GFP-LC3 cells transfected with control siRNA or Atg5 siRNA, were treated with IMB-6G (5 μM) for 6 h. The cells were fixed and analyzed for fluorescence microscopy. Original magnification: ×40. (**b**) GFP-LC3 puncta per cell were counted. Data are the mean ± SD for triplicate samples of at least 100 cells per sample. $^{*}p < 0.05$ compared with control group.

Supplementary Figure S3. IMB-6G selectively induced growth inhibition and apoptosis in pancreatic cancer cells. (**a**) MiaPaca-2, HupT-3 cells and normal

pancreatic ductal epithelial cell HPDE6-C7 cells were treated with 5μM IMB-6G for the indicated time, the growth inhibition of cells was measured by MTT assay. (**b**) MiaPaca-2, HupT-3 cells and HPDE6-C7 cells were treated with IMB-6G at the indicated concentrations for 24 h, and whole-cell lysates were subjected to immunoblotting with an anti-PARP1 antibody. PARP1 cleavage was used as an apoptosis marker.

a

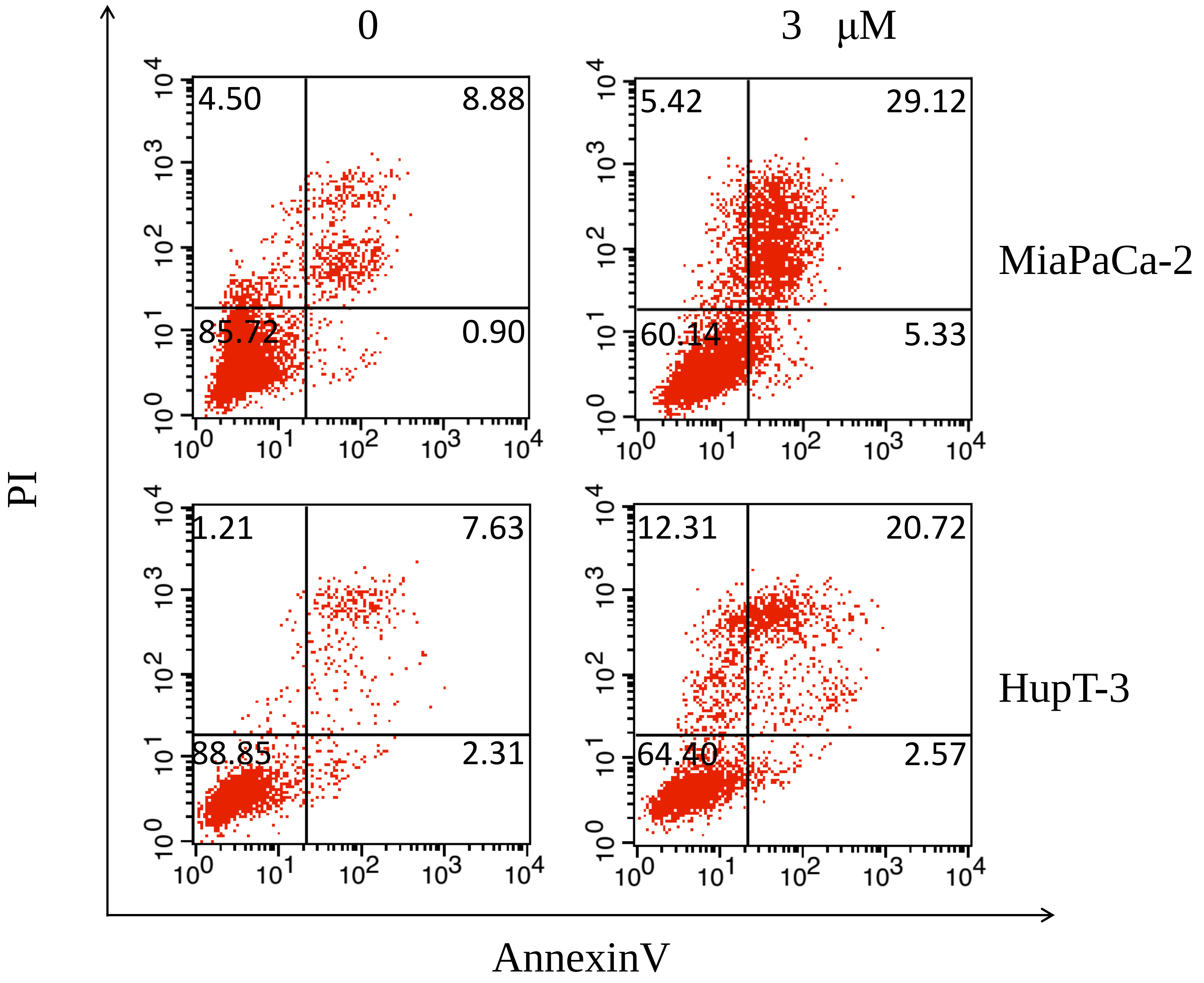
0
3 μM
PI
MiaPaCa-2
HupT-3
AnnexinV
4.50
8.88
85.72
0.90
5.42
29.12
60.14
5.33
1.21
7.63
88.85
2.31
12.31
20.72
64.40
2.57

b

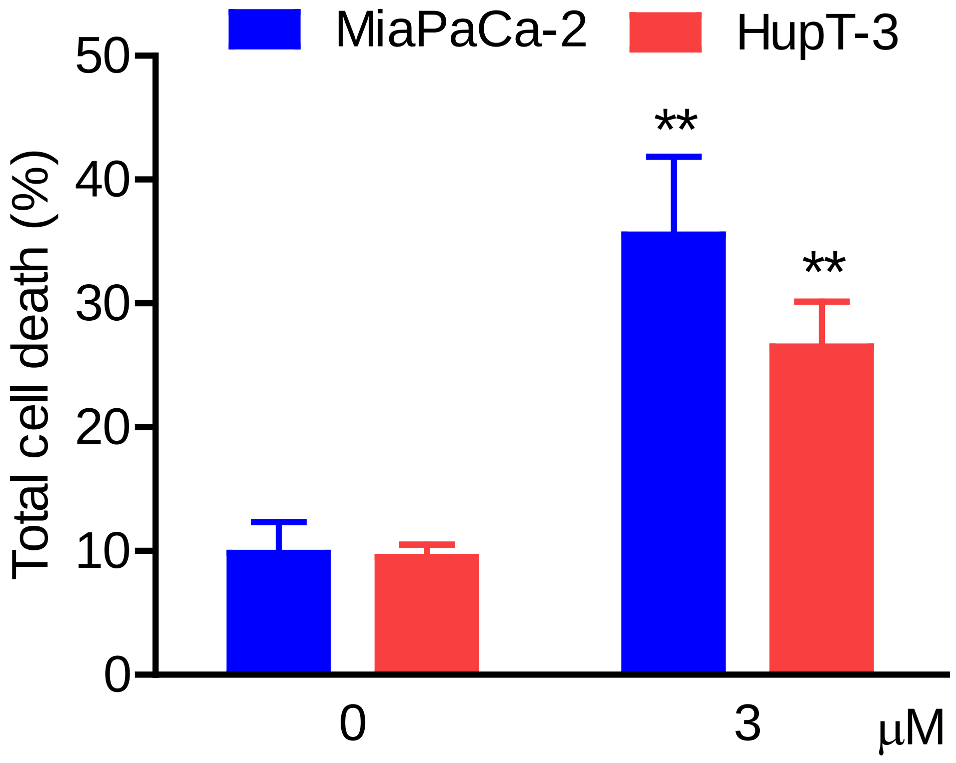
MiaPaCa-2
HupT-3
Total cell death (%)
**
**
0
3
μM

a

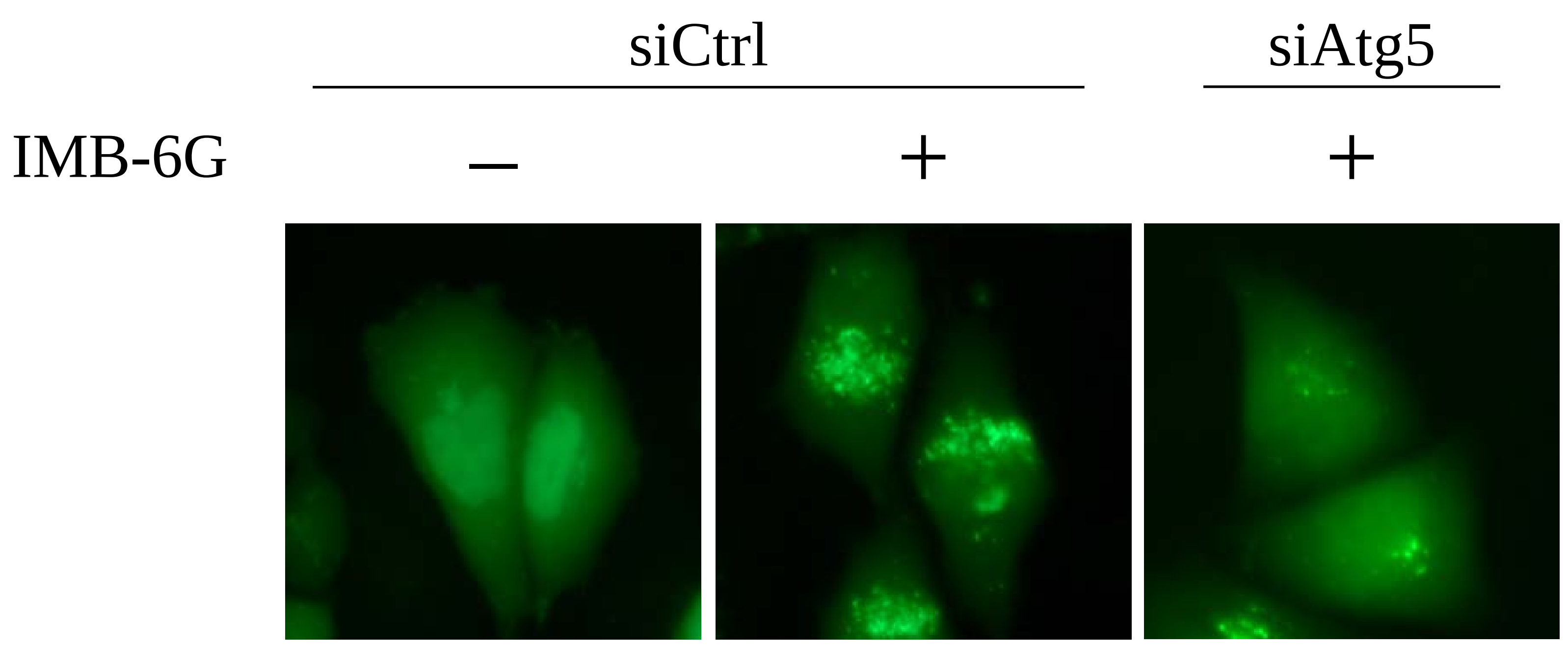
siCtrl
siAtg5
IMB-6G
–
+
+

b

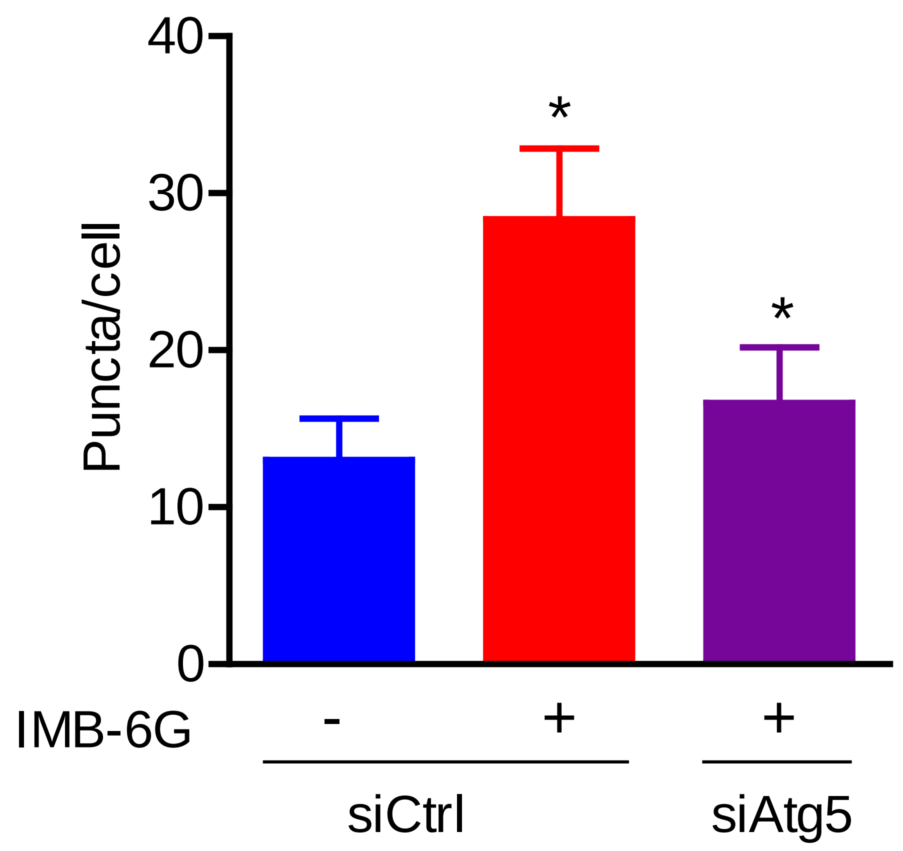
40
30
20
10
0
Puncta/cell
*
*
IMB-6G
-
+
+
siCtrl
siAtg5

a

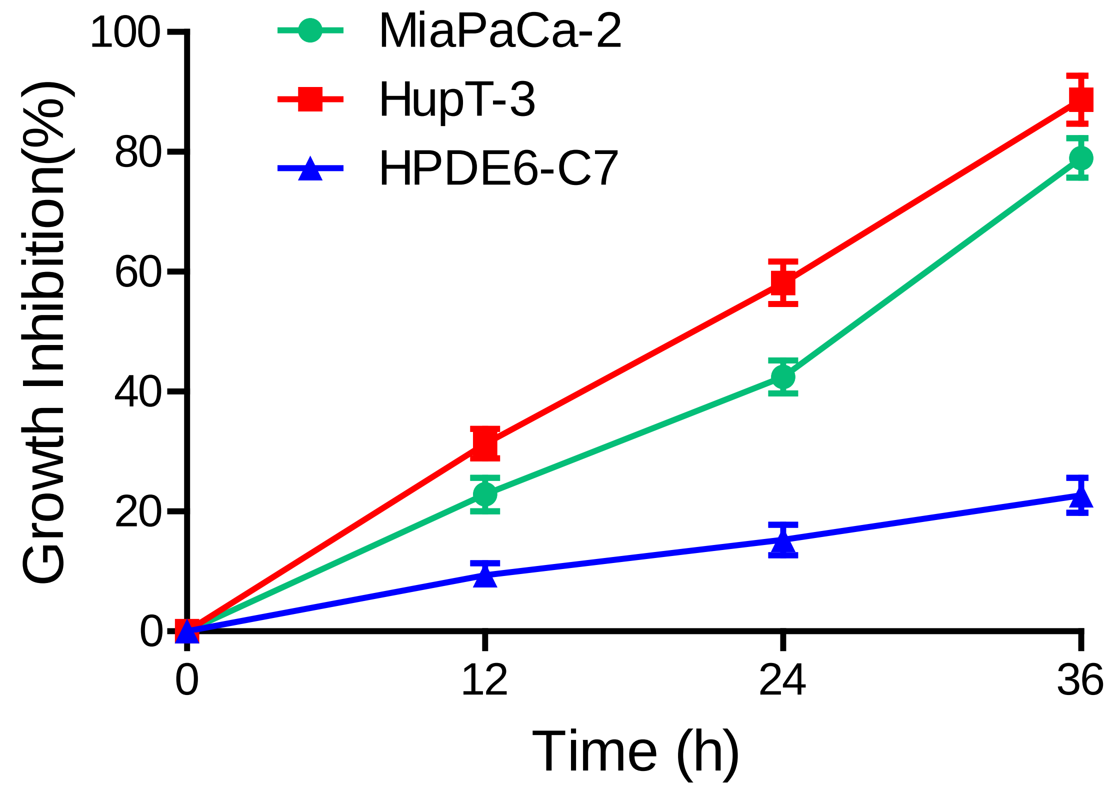
MiaPaCa-2
HupT-3
HPDE6-C7
Growth Inhibition(%)
100
80
60
40
20
0
0
12
24
36
Time (h)

b

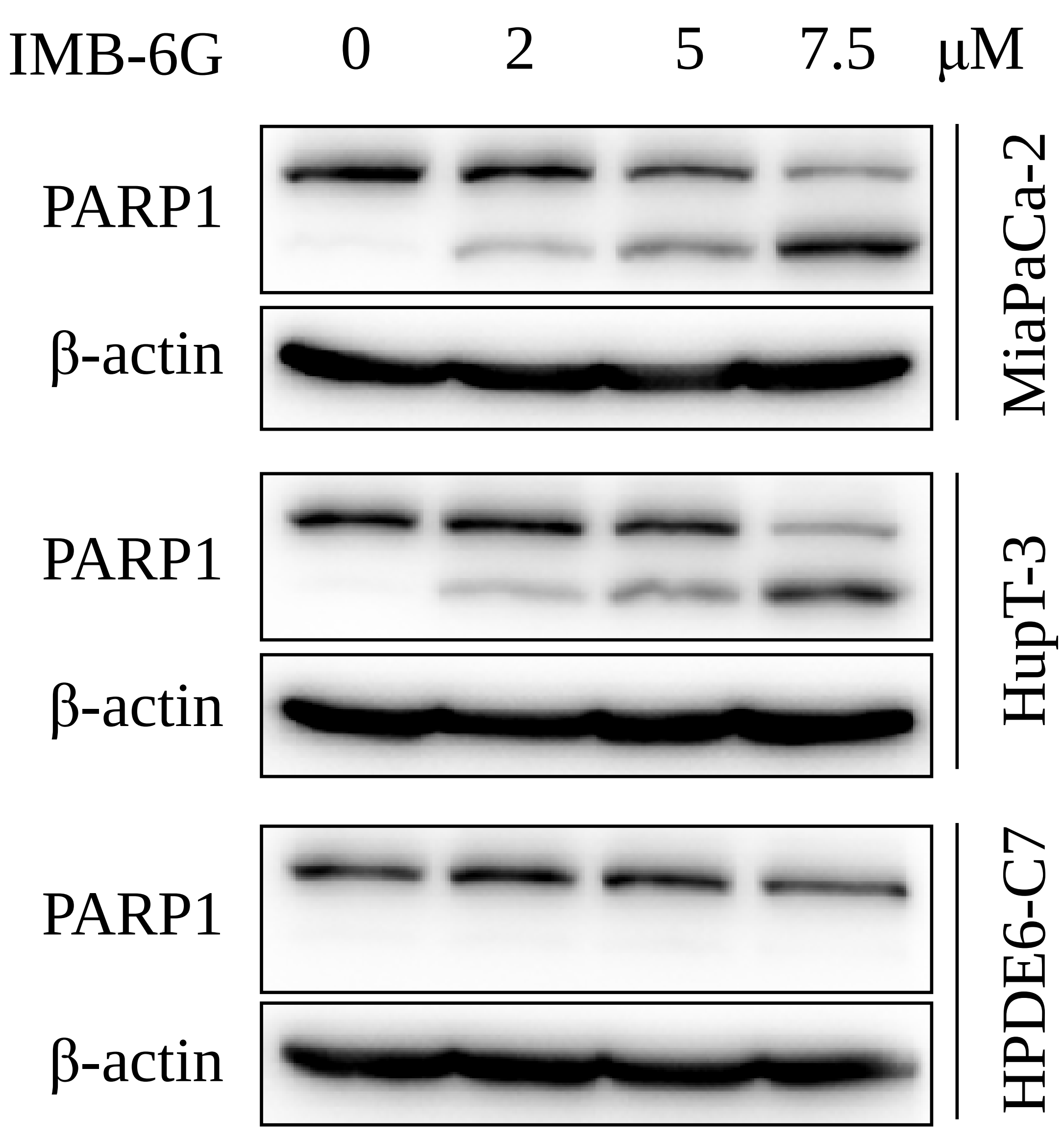
IMB-6G
0
2
5
7.5
μM
PARP1
β-actin
MiaPaCa-2
PARP1
β-actin
HupT-3
PARP1
β-actin
HPDE6-C7
